# Association between the intima-media thickness of the extracranial carotid arteries and metabolic syndrome in ethnic Kyrgyzs

**DOI:** 10.1186/s12872-018-0935-9

**Published:** 2018-10-22

**Authors:** Alina S. Kerimkulova, Olga S. Lunegova, Aibek E. Mirrakhimov, Saamay S. Abilova, Malik P. Nabiev, Ksenia V. Neronova, Erkaiym E. Bektasheva, Ulan M. Toktomamatov, Jyldyz E. Esenbekova, Erkin M. Mirrakhimov

**Affiliations:** 1 Kyrgyz State Medical Academy named after I.K. Akhunbaev, T.Moldo street 3, Bishkek, 720040 Kyrgyz Republic; 2 Kyrgyz Society of Cardiology, Bishkek, Kyrgyz Republic; 3 National Center of Cardiology and Internal Medicine named after academician M.M. Mirrakhimov, Bishkek, Kyrgyz Republic

**Keywords:** Carotid atherosclerosis, Intima-media, Carotid arteries, Metabolic syndrome, Kyrgyz

## Abstract

**Background:**

It is known that atherosclerosis is the leading cause of cardiovascular disease. We aimed to study the correlation between components of metabolic syndrome (MS) and subclinical carotid atherosclerosis in a group of ethnic Kyrgyzs.

**Methods:**

In а descriptive study we assessed 144 ethnic Kyrgyzs (69 males, 75 females) aged 36–73 years (average age 51.03 ± 8.2). All participants underwent a clinical investigation and an anthropometric evaluation (weight, height, waist circumference (WC)). Abdominal obesity (АО) was confirmed at WC ≥ 94 cm in males and ≥ 88 cm in females. Fasting plasma glucose and lipid spectrum tests were performed. An ultrasound assessment of carotid intima-media thickness (IMT) was performed using a 7.5 MHz transducer (Phillips-SD 800).

**Results:**

MS was revealed in 61 (42.4%; 47.8% in men and 37.3% in women) of the investigated patients. IMT was significantly increased with the presence of MS components in males (no components vs 2 components of MS: 0.67 ± 0.007 and 0.81 ± 0.009 respectively; р < 0.05) and females (no components vs 3 components of MS: 0.63 ± 0.007 and 0.76 ± 0.01 respectively; р < 0.01). IMT trended towards an increase in the presence of a greater number of MS components in patients with and without AO (*р* < 0.01). In order to identify independent factors affecting IMT we carried out a multifactorial logistic regression analysis. Arterial hypertension was found to have the greatest influence on the development of MS (OR = 3.81, *p* < 0.0001).

**Conclusion:**

In the group of ethnic Kyrgyzs, a greater number of MS components, with AO or without AO, is associated with higher carotid IMT.

## Background

It is known that atherosclerosis is the leading cause of cardiovascular disease (CVD), accompanied by increased mortality and disability [1]. Therefore, it is important to identify people at high risk of CVD at the earliest stage. Indeed, the presence of more than one atherosclerosis risk factors significantly aggravates the overall CVD risk. Based on this concept, metabolic syndrome (MS) has been highlighted as a cluster of risk factors for atherosclerosis.

An accessible method for estimating pre-clinical atherosclerosis is thickness of the carotid intima-media complex (CIMT), which is measured using non-invasive ultrasound scanning [2]. At the same time, CIMT is an important predictor of coronary atherosclerosis [3, 4]. Along with coronary atherosclerosis, studies demonstrated a gender correlation between IMT and metabolic syndrome [5]. However, as has been shown in epidemiological studies, the correlation between CIMT and MS was identified based on the Western population, or by using the MS criteria for Adult Treatment Panel (ATP) III [6, 7].

It should be emphasized that there have been no studies carried out on the relationship between CIMT and MS in ethnic Kyrgyz. Moreover, it is interesting to know whether each component of MS equally contributes to an increase in CIMT, and which MS components have the strongest association with the increase in CIMT [6, 8, 9].

The purpose of this research is to study the correlation between MS components and sub-clinical carotid atherosclerosis in a group of ethnic Kyrgyz.

## Methods

**Fig. 1:**
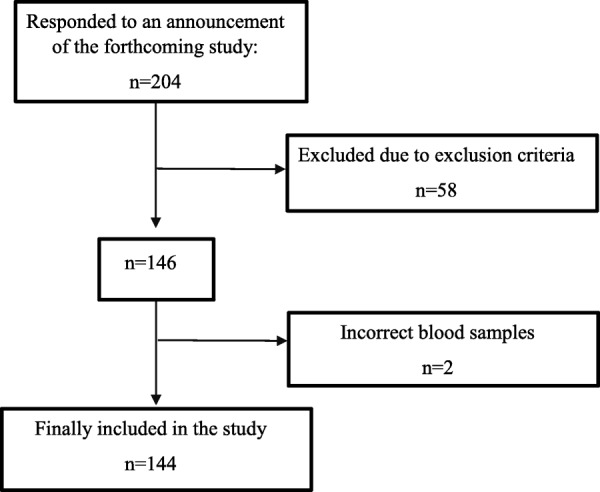
The flow chart of the study

The research included the ethnic Kyrgyz over 35 years of age, who are residents of the Kyrgyz Republic, and who responded to an announcement of the forthcoming study. This study excluded patients with severe chronic liver, kidney or thyroid dysfunction, as well as people who received corticosteroids or insulin, and pregnant and lactating women. The flowchart of the study is presented in Fig. 1.

The patients underwent a clinical examination including an assessment of complaints and anamnesis, as well as an objective examination with the measurement of anthropometric parameters including height, weight, waist circumference (WC), and systolic (SBP) and diastolic (DBP) blood pressure (BP). The average BP was calculated as the arithmetic mean of SBP and DBP. The body mass index (BMI) was calculated using the formula: BMI = weight (kg)/height (m)2. Obesity was established at BMI ≥30 kg/m2.

The laboratory tests included the blood plasma analysis of glucose (fasting), the lipid spectrum (total cholesterol (TC), triglycerides (TG) and high-density lipoprotein cholesterol (HDL-C). The blood samples were collected and centrifuged, then the serum was separated and frozen at − 20 °C. All the biochemical analyses were conducted at the Dir Adjoint du Département Hommes, Natures, Musée de l’Homme (Paris, France). Low-density lipoprotein cholesterol (LDL-C) was calculated according to Friedwald’s formula [10].

### The measurement of CIMT

The sub-clinical and structural changes in the extracranial section of the right and left common carotid arteries were evaluated using a 7.5 MHz linear vascular sensor (echocardiograph Phillips-SD 800). The measurement of CIMT was performed in the middle third of the common carotid artery, along with the back wall of the vessel, and in the areas free of atherosclerotic plaques. CIMT was evaluated based on systole and diastole, then the obtained data were averaged. For calculations, the arithmetic mean of the left and right carotid arteries were used. The measurements of carotid parameters were evaluated in accordance with the criteria of the European Carotid Surgery Trialists 1991 [11].

### The definition of MS

MS was diagnosed using the modified criteria that include the presence of abdominal obesity (AO) and two or more of the following conditions: arterial hypertension (AH), dyslipidemia and hyperglycemia [12]. For AO, the following values were taken from the Kyrgyz average WC of ≥94 cm in men and ≥ 88 cm in women [13]. AH was established at SBP ≥130 mmHg or at DPB ≥ 85 mmHg, or in patients taking antihypertensive drugs. Dyslipidemia was established at a TG level of 1.7 mmol/L and/or HDL-C < 1.03 in men and < 1.29 mmol/L in women, or in patients using lipid-lowering drugs. Hyperglycemia was determined at a fasting glucose level > 5.6 mmol/L, or in patients receiving treatment for type 2 diabetes mellitus (DM) [14].

The statistical analysis was carried out with the aid of STATISTICA 7.0 (StatSoft Inc., USA). The variable distribution was analyzed using the Kolmogorov-Smirnov test. The variables with normal and non-parametric distributions are presented as a mean ± standard deviation and median (25th–75th percentiles), respectively. The differences in the characteristics of patients with MS and without MS were analyzed using the Student’s t-test for parametric variables and the Mann-Whitney test for non-parametric variables. Furthermore, a comparison of the groups by their binary features was carried out by the χ2 test. The effect of MS and the increase in the number of MS components in CIMT was assessed by the single-factor parametric variance analysis (ANOVA). The a posteriori group comparison was performed by post-hoc analysis with the Bonferroni amendment. In order to identify independent factors impacting on CIMT, we carried out a multifactorial logistic regression analysis to find out which MS component is significantly associated with an elevated level of IMT. In particular, values from 75 percentiles of IMT and higher were considered as the elevated level of IMT. The independent variables included gender, age, arterial hypertension, level of glycemia and triglycerides. Were conducted by a post-hoc sample size calculation was performed to estimate the sampling size (by using the calculator available at http://clincalc.com/stats/SampleSize.aspx).

The criterion for statistical significance was set at *p* < 0.05.

## Results

One hundred and forty-four (144) ethnic Kyrgyzs (69 men and 75 women) aged 36 to 73 years were examined; the average age of the patients was 51.03 ± 8.2 years (for men: 51.9 ± 8.7 years, for women: 50.2 ± 7.7 years).

We conducted the post-hoc sample size calculation. According to the results of the sample size calculation, 120 patients (60 in both groups) are required to have an 80% chance of detecting, as significant at the 5% level. The sample size required per group – 60. The total sample size required – 120 (Alpha – 0.05; Beta – 0.2; Power – 0.8). The subgroups (men and women, with and without metabolic syndrome): men: the sample size required per group – 34. The total sample size required – 68 (Alpha – 0.05; Beta – 0.2; Power – 0.8). Women: the sample size required per group – 25. The total sample size required – 50 (Alpha – 0.05; Beta – 0.2; Power – 0.8).

MS was detected in 61 (42.4%) of the examined patients (47.8% of men and 37.3% of women). Table 1 presents the clinical and biochemical characteristics of patients depending upon whether they had MS or not. In patients with MS, there were large values of BMI, WC, SBP, DBP, TG, blood glucose and a lower level of HDL-C. Besides, the women with MS were older than those without MS, whilst the men were of a comparable in age (Table 1).

**Table 1:** Characteristics of the examined patients, depending on the presence of metabolic syndrome

	Men		Women	
	MS not present (*n* = 36)	MS present (*n* = 33)	MS not present (*n* = 47)	MS present (*n* = 28)
Age	52.3 ± 9.5	51.6 ± 8.0	48.6 ± 7.2	52.9 ± 7.9*
BMI, kg/m^2^	26.4 ± 3.1	29.8 ± 3.7^$^	26 ± 4.5	31.3 ± 4.5^
WC, cm	93.8 ± 8.5	103.5 ± 8.3^$^	83.4 ± 9.5	96.8 ± 6.5^
SBP^#^, mmHg.	135 (128–152)	146 (135–157)*	128 (119–136)	140 (134–160)^**&**^
DBP^#^, mmHg.	89 (81–96)	93 (89–102)**	83 (77–91)	91 (80–96)
TC, mmol/L	5.1 ± 0.9	5.5 ± 0.9	5.02 ± 0.9	4.97 ± 1.3
TG^#^, mmol/L	1.2 (0.9–1.4)	2.2 (1.7–3.5)^	1.0 (0.8–1.2)	1.5 (1.1–2.0)^$^
HDL-C^#^, mmol/L	1.15 (1.02–1.4)	0.83 (0.7–1.0)^	1.4 (1.3–1.6)	1.03 (0.8–1.2)^
LDL-C, mmol/L	3.2 ± 0.8	3.4 ± 0.9	3.1 ± 0.8	3.2 ± 1.03
Glucose^#^, mmol/L	5.2 (5.04–5.4)	6.2 (5.7–6.6)^	5.2 (4.9–5.5)	5.8 (5.5–6.3)^
Smoking, n (%)	15 (41.7)	11 (33.3)	0 (0)	0 (0)
AH, n (%)	18 (50)	27 (81.8)*	12 (25.5)	18 (64.3)**
Dyslipidemia, n (%)	12 (33.3)	32 (96.7)**^**	14 (29.8)	27 (96.4)**^**
Hyperglycemia**,** n (%)	5 (13.9)	25 (75.8)**^**	7 (14.9)	20 (71.4)**^**
IMT^#^, mm	0.72 ± 0.01	0.78 ± 0.01	0.66 ± 0.009	0.72 ± 0.01*

*BMI* body mass index, *WC* waist circumference, *SBP* systolic blood pressure, *DBP* diastolic blood pressure, *TC* total cholesterol, *TG* triglycerides, *HDL-C* cholesterol of high-density lipoproteins, *LDL-C* low-density lipoprotein cholesterol. Hereinafter in Tables 2 and 3: *AH* arterial hypertension, *MS* metabolic syndrome, *IMT* the average thickness of the intima-media complex; # = the data are represented as median (25–75%), * - *p* < 0.05; ** - *p* < 0.01; & - *p* < 0.001; $ - *p* < 0.0001; ^ - *p* < 0.00001

We analyzed the pharmacological agents taken by patients (see Table 2). In men, there were no statistically significant differences in the medication use in the groups with and without MS. Among women, those with MS in comparison to women without MS were more likely to take ACE inhibitors (28.6% and 8.5% respectively, *p* < 0, 05). There were no statistically significant differences in other groups by use of medications (Table 2). All patients with diabetes mellitus were managed with glibenclamide, but none was on metformin. There were no statistically significant differences between the subgroups with and without MS (Table 2). The patients in both groups did not take statins. After obtaining the results of the lipid spectrum, the patients were recommended to take statins, as well as the recommendations were provided for correcting cardiometabolic risk factors.

**Table 2:** Characteristics of medications use depending on the presence or the absence of metabolic syndrome

Parameters	Men		Women	
	MS + (*n* = 36)	MS - (*n* = 33)	MS + (*n* = 47)	MS - (*n* = 28)
ACEI, n (%)	5 (15,2)	3 (8,3)	8 (28,6)	4 (8,5)*
Amlodipine, n (%)	0 (0)	1 (2,8)	1 (3,6)	1 (2,1)
Other Ca antagonists (Verapamil, Nifedipine), n (%)	1 (3,03)	0 (0)	1 (3,6)	0 (0)
Beta blockers (atenolol), n (%)	3 (9,1)	2 (5,6)	1 (3,6)	3 (6,4)
Indapamide, n (%)	1 (3,03)	0 (0)	0 (0)	0 (0)
Oral glucose lowering drug (glibenclamide), n (%)	2 (6,1)	0 (0)	1 (3,6)	0 (0)

*ACEI* angiotensin converter enzyme inhibitors, *MS* metabolic syndrome; * - *p* < 0.05 in women

A comparison of the CIMT values depending on the presence or absence of MS and the number of MS components is shown in Table 3. Taking into account that the studies have shown that carotid IMT in men differs from IMT in women [15], we analysed IMT separately for each gender.

**Table 3:** IMT depending on the presence or absence of metabolic syndrome and the number of components

	Men		Women	
	n	IMT. mm	n	IMT. mm
MS not present	36	0.72 ± 0.01	47	0.66 ± 0.009
MS present	33	0.78 ± 0.01	28	0.72 ± 0.01
		*р* = 0.07		*р* < 0.05
Number of MS components				
0	10	0.67 ± 0.007	18	0.63 ± 0.007
1	20	0.72 ± 0.01	28	0.68 ± 0.009
2	18	0.81 ± 0.009*	17	0.69 ± 0.01
3	21	0.76 ± 0.01	12	0.76 ± 0.01*^#^
		*р* < 0.05		*р* < 0.01

* - *p* < 0.01 - in comparison with patients without a single MS component; # - *p* < 0.05 in comparison with patients with two components of MS

In both sexes, there was a tendency for an increase in CIMT in persons with MS compared to those without MS. Moreover, in women, this trend was statistically significant (*p* < 0.05). All patients were divided up into four groups based on the number of MS components: group 1 in which the patients did not have any component of MS; groups 2–4 included patients with the presence of one to three components of MS: AH, dyslipidemia and hyperglycemia, respectively. In both men (p < 0.05) and in women (*p* < 0.01), a gradual increase was observed as the number of MS components increased. In addition, CIMT in men with two components of MS was significantly greater than in patients without a single component of MS. The women with three MS components had greater CIMT than the patients with two and without a single MS component (Table 3).

**Fig. 2:**
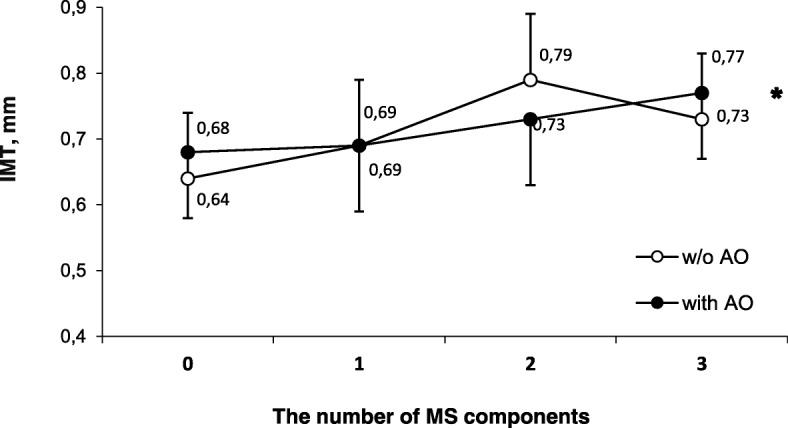
IMT in the carotid artery depending on the presence or absence of abdominal obesity. Notes: IMT - intima-media complex thickness; AO - abdominal obesity; MS - metabolic syndrome; the data are presented as mean ± standard deviation; * *p* < 0.01

The effect of the increase in the number of MS components in IMT was analyzed depending on the presence or absence of AO (Fig. 2). In patients with or without AO, there was a tendency for an increase in IMT as the number of MS components increased. At the same time, in the cases where persons did not have AO, this tendency was statistically significant (p < 0.01) (Fig. 2).

To determine the independent association of MS components with carotid IMT, a multiple regression analysis was performed (Table 4). In men age (β = 0.523, *p* < 0.00001), and in women age (β = 0.354, *p* < 0.001) and the mean BP (β = 0.369, p < 0.001) were significantly associated with carotid IMT.

**Table 4:** Logistic regression analysis with the dependent variable – increased IMT value

	Controlling for age, sex, AH, serum glucose, TG		
	OR	95 CI	p<
Male sex	0.42	0.44–0.60	0.0001
Age	1.13	49.67–52.38	0.0001
AH	3.81	0.44–0.60	0.0001
Glucose, mmol/l	1.21	5.56–6.17	0.0001
TG, mmol/l	1.23	1.38–1.73	0.0001

*AH* arterial hypertension, *TG* triglycerides

## Discussion

After having analyzed the results of the 144 ethnic Kyrgyzs, we found that the combination of MS components affects CIMT. Amongst the components of MS, the factors that had the strongest associations with CIMT were arterial hypertension and AO.

The effect of an increase in the number of the MS components on carotid atherosclerosis has been shown in some epidemiological studies [6, 16, 17]. In our work, we also found an increase in IMT as the number of MS components increased in patients of both sexes.

Nevertheless, in the analysis of IMT depending on the number of components of MS and AO, the association between IMT and MS was not statistically significant. In this case, we showed that IMT in patients with AO and without AO was similar with the same number of MS components. In other studies [18], a more frequent occurrence of carotid atherosclerosis was observed in persons with a large number of MS components, regardless of the presence of AO. Moreover, Lee et al. [19] showed the similar risk of developing coronary heart disease in patients with both AO and without AO. These results suggest that the central type of obesity is not always indicative of an increased risk of CVD.

It is known that AO is a significant predictor of insulin resistance, which, in turn, leads to impaired glucose tolerance, hypertension and dyslipidemia [20]. The results obtained by us suggest that AO may not be the immediate cause of atherosclerosis. However, AO is included in a cluster of risk factors, including AH, dyslipidemia, and hyperglycemia. The research has shown that AO has a key position in the set of risk factors associated with the development of atherosclerosis [21].

In our study, CIMT was associated with MS, but a relationship with AO could not be identified. However, it is not prudent to ignore patients with normal WC, but with a combination of other components of MS, since such patients still, have a risk of further worsening IMT.

In the present work, it was initially planned to determine whether the MS components were equally associated with IMT. We examined the effect of each component of MS on IMT and found that, in patients with AO, in contrast to those with dyslipidemia and hyperglycemia, there were significantly higher rates of IMT than in patients without a single MS component. These results suggest that not all components of MS have a similar atherosclerotic risk. The most important determinants contributing to the development of thickening of IMT appear to be AH and age.

AH is recognized as an important risk factor for the development of atherosclerosis, CVD, and strokes. In a study by Ishizaka et al. [22], it was shown that among the five components of MS according to the ATP III criteria, carotid atherosclerosis was strongly associated with AH. Furthermore, Su et al. [23], studied risk factors such as hypertension, hypercholesterolemia, hypertriglyceridemia and type II diabetes, and recognized AH as the most significant risk factor for increased IMT and the development of carotid stenosis.

In the present study, we found that arterial hypertension has the highest association with CIMT. At the same time, some population-based studies have confirmed an association between CIMT and AH as well as other traditional risk factors for atherosclerosis such as smoking, dyslipidemia and hyperglycemia [24, 25]. Furthermore, the studies have shown that the risk of MS is not always determined by the number of its components in an individual. Thus, we have shown significant influence on IMT clusters by different components of MS, whilst the composition of clusters consistently included elevated blood pressure [26, 27]. Although hypertension is a significant risk factor for increasing CIMT, we should not ignore individuals with other risk factors.

We believe that every component of MS including AH, dyslipidemia, and hyperglycemia is a risk factor for increasing СIMT. At the same time, the effect of MS components on CIMT can be uneven. This study shows that patients with AH probably have a higher risk of carotid arteriosclerosis than patients without AH. The presence of hypertension amongst MS components in an individual suggests that additional preventative approaches are used in such patients.

It ought to be mentioned that there are some limitations to interpreting the results of this study. Firstly, the examined patients may not meet strict criteria for the representativeness of the Kyrgyz population as a whole. We included patients who responded to the announcement of the forthcoming study, so there could be the possibility (or there was room for) of a systematic error in the selection process. In this study, the incidence of MS was slightly higher than in previous studies of ethnic Kyrgyzs [28], and, the prevalence of MS was higher than in the studies of Europeans [7]. Secondly, the present results were obtained in a cross-sectional study, which does not allow us to conclude the temporal sequence of the observed association. In this regard, prospective studies are needed to assess the long-term effects of MS on CIMT. Thirdly, it is necessary to note that, a relatively small number of patients was included in the study. However, the post-hoc sample size calculation showed that the included number of patients was sufficient. Fourthly, the ultrasound of carotid arteries was conducted once by a single provider blinded to the study. We did not study endothelial function, because it did not fall within the primary study objective. Nonetheless, the determination of endothelial function is also crucial for the early diagnosis of atherosclerosis [29, 30].

## Conclusion

This study shows that an increase in the number of components of MS, with or without AO, is associated with greater CIMT. We also found that the risk of CIMT differs among MS components. The most important determinants of CIMT were age and AH. Our results support the view that simply diagnosing MS is not sufficient to establish risk factors for atherosclerosis in an individual and recommend a qualitative and quantitative assessment of multiple components of MS.

## Abbreviations

AH: Arterial hypertension
AO: abdominal obesity
ATP: Adult Treatment Panel
BMI: Body mass index
BP: Blood pressure
CIMT: Thickness of the carotid intima-media complex
CVD: Cardiovascular disease
DBP: Diastolic blood pressure
DM: Diabetes mellitus
HDL-C: High-density lipoprotein cholesterol
IMT: Intima-media thickness
LDL-C: Low-density lipoprotein cholesterol
MS: metabolic syndrome
SBP: systolic blood pressure
TC: total cholesterol
TG: triglycerides
WC: waist circumference
